# Kuhuang injection exerts a protective effect by activating PPAR-γ in an *in vitro* model of chlorpromazine-induced cholestatic liver injury constructed by tissue engineering

**DOI:** 10.1080/13880209.2022.2110128

**Published:** 2022-09-05

**Authors:** Qiao Wu, Zhongping Duan, Long Huang, Zhijie Li

**Affiliations:** a Infection Center, Beijing Tiantan Hospital, Capital Medical University, Beijing, China; b Fourth Department of Liver Disease (Difficult & Complicated Liver Diseases and Artificial Liver Center), Beijing You’an Hospital Affiliated with Capital Medical University; Beijing Municipal Key Laboratory of Liver Failure and Artificial Liver Treatment Research, Beijing, China; c Hepatobiliary Surgery Center, The Fifth Medical Center of PLA General Hospital, Beijing, China

**Keywords:** Drug-induced cholestasis, tissue-engineered liver, Chinese medicine, antioxidation

## Abstract

**Context:**

Kuhuang (KH) injection is a widely used anticholestatic drug in the clinic and the mechanisms are still unclear.

**Objective:**

This study uses a new 3D tissue-engineered (TE) liver platform to study the ability of kuhuang to ameliorate liver injury induced by chlorpromazine (CPZ) and the possible mechanisms involved.

**Materials and methods:**

The TE livers (*n* = 25) were divided into 5 groups (*n* = 5 livers/group) as 3D, 3D + CPZ, 3D + CPZ + KH, 3D + CPZ + GW9662 (a PPARγ inhibitor) and 3D + CPZ + KH + GW9662. The treatments with kuhuang (1 mg/mL) and GW9662 (10 μmol/L) were given to the desired groups on the 7th day of the experimental process. 20 μmol/L CPZ was added on the 8th day.

**Results:**

According to the 2D experimental results, the minimum effective concentration of kuhuang is 10 μg/mL and the optimal effective concentration is 1 mg/mL. Kuhuang ameliorated tissue damage in the TE livers both in terms of tissue structure and culture supernatant. Kuhuang significantly reduced TBA accumulation (38%) and downregulated CYP7A1 (38%) and CYP8B1 (79%). It reduced hepatic levels of ROS (14%), MDA (27%) but increased the levels of GSH (41%), SOD (12%), BSEP (4.4-fold), and MRP2 (74%). Moreover, kuhuang downregulated DR5 (99%) but increased the mRNA expression of PPARγ (4-fold). Molecular docking analyses determined the bioactivity of the active compounds of kuhuang through their specific bindings to PPARγ.

**Conclusions:**

Kuhuang could alleviate CPZ-induced cholestatic liver injury by activating PPARγ to reduce oxidative stress. Applying kuhuang for the treatment of CPZ-induced liver injury could be suggested.

## Introduction

Drug-induced liver injury (DILI) is a common clinical adverse drug reaction and an important factor in drug development failure and withdrawal (Kullak-Ublick et al. 2017). Cholestatic injury and mixed hepatocytic/cholestatic injury constitute the two main subtypes of DILI and may account for 50% of all DILI cases (Shen et al. 2019). Drug-induced cholestasis (DRIC) may manifest clinically as pruritus, malaise, darkened urine, and jaundice and is often asymptomatic in the early stages, eventually manifesting as elevated serum alkaline phosphatase (ALP) and γ-glutamyl transpeptidase (GGT) levels and possibly progressing to hyperbilirubinemia, which can lead to liver failure or even death in severe cases (Padda et al. 2011). At present, effective drugs for the clinical treatment of cholestasis are lacking. Ursodeoxycholic acid (UDCA) and obeticholic acid (OCA) are the clinically approved drugs for cholestasis and are recognized to have certain curative effects, but their therapeutic effect is limited, as there are large individual differences in their effects (Santiago et al. 2018). Thus, the treatment strategies for DRIC are not ideal. Therefore, it is necessary to develop new drugs for the treatment of cholestatic liver injury. Furthermore, traditional Chinese medicine has received increasing attention in the treatment of DRIC (Li et al. 2019; Wang et al. 2020; Hua et al. 2021).

Kuhuang injection is a traditional Chinese medicine injection made from traditional Chinese medicinal materials such including Radix Bupleuri, Sophora Flavescens, Rheum Officinale, Herba Artemisiae, and Folium Isatidis (shown in Table 1), which are extracted and refined through modern technologies (Wu et al. 2005a, 2005b). It has the effects of clearing heat, removing dampness, soothing the liver, and relieving jaundice. It is commonly used clinically to relieve jaundice and protect the liver. Clinical reports have revealed that it protects the liver and ameliorates intrahepatic cholestasis, but the specific mechanism by which it alleviates jaundice is still unclear (Xu et al. 2009).

**Table 1.** Raw material information of kuhuang injection.

Latin names of medicinal herbs	Latin name of family	Latin name of plant
Artemisiae Scopariae Herba	Compositae	*Artemisia scoparia* Waldst.et Kit. *Artemisia capillaris* Thunb.
Bupleuri Radix	Umbelliferae	*Bupleurum chinense* DC. *Bupleurum scorzonerifolium* Willd.
Rhei Radix et Rhizoma	Polygonaceae	*Rheum palmatum* L. *Rheum tanguticum* Maxim. ex Balf. *Rheum officinale* Baill.
Sophorae Flavescentis Radix	Leguminosae	*Sophora flavescens* Ait.
Isatidis Folium	Brassicaceae	*Isatis indigotica* Fort.

Note: The name of the herb include the medicinal part, such as Herba for whole herb, Radix for root, Rhizoma for rootstock, Folium for leaf. Composition and format of Binomial Nomenclature: Genus name (italic, initial letter capitalized) + type plus words (italic, all lowercase letters) + species namer's name (orthographic, initial letter capitalized).

Information source: Chinese Pharmacopoeia 2020 Edition.

The mechanisms of DRIC are diverse, and some of the related factors are unknown (Sundaram and Björnsson 2017; Chatterjee and Annaert 2018). Changes in the expression of liver bile transport proteins (BSEP and MRP2) in addition to other mechanisms, such as damage to the cytoskeleton and oxidative stress, contribute to the pathological process of cholestasis (Anthérieu et al. 2013; Hendriks et al. 2016; Burban et al. 2018). CPZ is a neuroleptic phenothiazine. It is widely used in the clinical treatment of schizophrenia and has been associated with several cases of hepatic injury, mainly intrahepatic cholestasis (Todorović Vukotić et al. 2021). However, its initial toxic effects are still largely ignored, possibly because the current models used for safety assessment in drug development cannot accurately predict human cholestasis. Therefore, it is necessary to construct a new preclinical cellular model of DRIC. Because the source of fresh human hepatocytes is limited, we used differentiated human HepG2 cells expressing phase I and phase II drug-metabolizing enzymes and transporters (Hewitt and Hewitt 2004; Ramaiahgari et al. 2014) and established a novel three-dimensional (3D) TE liver platform *in vitro* by recellularization of a naturally obtained rat decellularized scaffold (Wu et al. 2018; Han et al. 2019; Zhang et al. 2021) in this study. By using this platform, we constructed a DRIC model by adding CPZ (20 μmol/L) and bile acid mixtures (shown in Table 2) to analyse the characteristics of intrahepatic cholestasis induced by CPZ treatment and to explore the protective effect of kuhuang injection against CPZ-induced cholestasis.

**Table 2.** Composition of the bile acid mixture and the concentrations.

Bile acid	Concentration (μM)
Cholic acid	0.41
Chenodeoxycholic acid	0.64
Deoxycholic acid	0.48
Lithocholic acid	0.008
Ursodeoxycholic acid	0.14
Glycylchenodeoxycholic acid	0.80
Total	2.478

## Materials and methods

DMEM was purchased from Thermo Fisher Biochemicals Co., Ltd. (USA). Foetal bovine serum, PLA2 enzyme, and penicillin were purchased from Gibco (USA). An AMV one-step RT-PCR kit was obtained from TaKaRa (China). Kuhuang injection extract powder was provided by Changshu Leiyunshang Pharmaceutical Co., Ltd. (China). A peristaltic pump was purchased from Master-Flex (German), and a 0.22 μm filter (Millex-GV) (German) was used. Sodium Deoxycholate (SDC) was obtained from VETEC (USA), and disposable indwelling intravenous needles (BD type Y 22 G) (USA) were used. A cell counting kit-8 (CCK-8) kit was purchased from Dojindo (USA), and reactive oxygen species (ROS) detection kits were purchased from Shanghai Biyuntian (China). Malondialdehyde (MDA) assay kits, superoxide dismutase (SOD) assay kits, glutathione (GSH) assay kits, and TBA assay kits were purchased from Nanjing Jiancheng Institute of Bioengineering (China). Aspartate transaminase (AST) test kits, alanine transaminase (ALT) test kits, lactate dehydrogenase (LDH) test kits, and alkaline phosphatase (ALP) test kits were all obtained from Nanjing Jiancheng Institute of Biological Engineering (China). CPZ was purchased from MCE (MedChemExpress) (USA), and GW9662 was obtained from Selleck (USA).

## Animals

Male Sprague-Dawley (SD) rats (body weight: 180–220 g) were obtained from the Weitong Lihua Experimental Animal Centre. The animals were bred normally before the experiment. The Capital Medical University Animal Experiments and Experimental Animals Management Committee (AEEI-2020-076) approved all protocols for feeding, anaesthesia, blood and tissue sampling, and euthanasia of animals.

### Preparation of decellularized liver scaffolds

**Figure 1.**
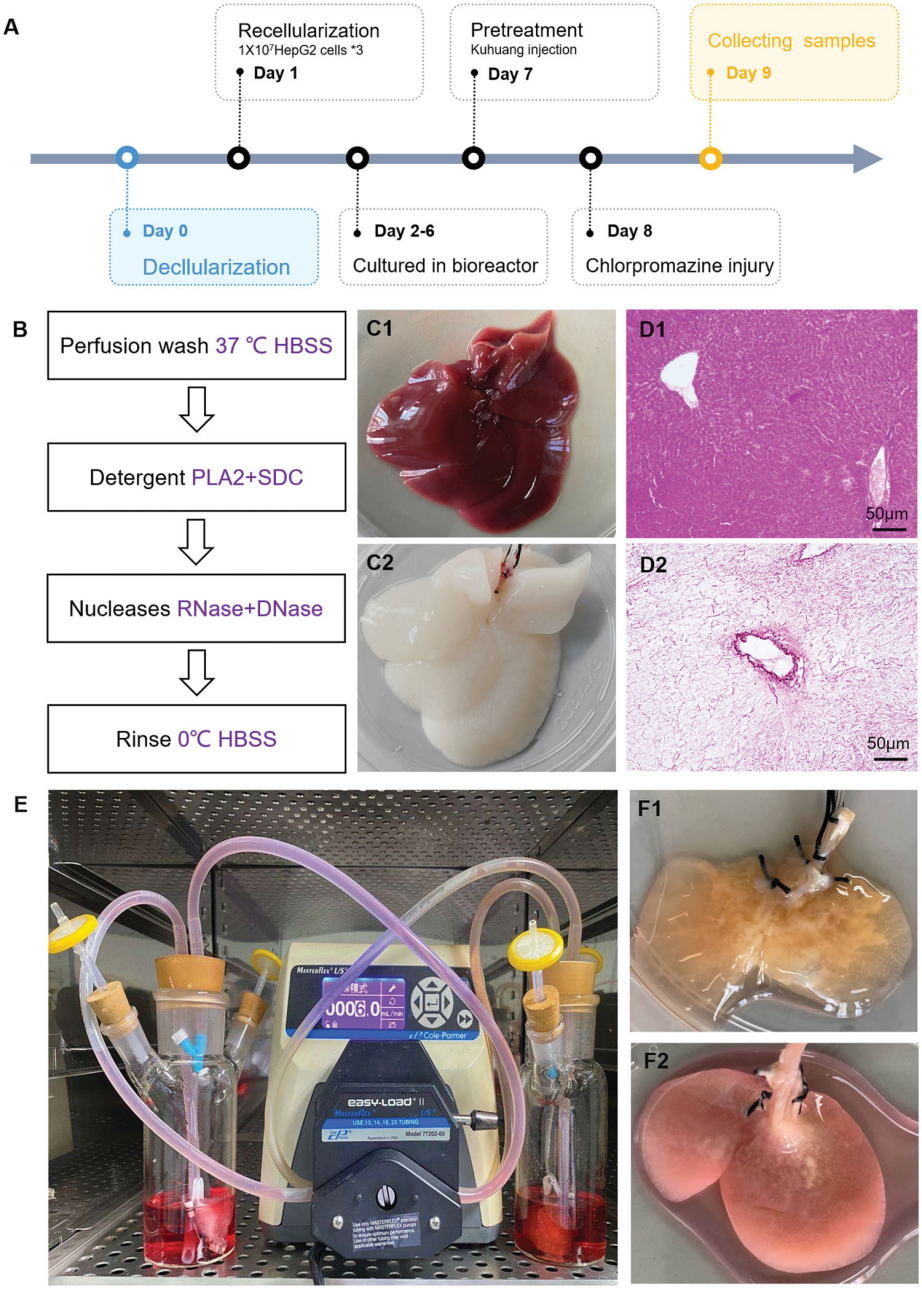
Constructing *in vitro* model of chlorpromazine-induced cholestatic liver injury by tissue engineering. (A) Schematic diagram of the model establishment process. (B) The process of obtaining decellularized scaffolds. (C) Appearance of normal rat liver (C1) and decellularized rat liver scaffold (C2). D. HE staining of normal rat liver (D1) and decellularized rat liver scaffold (D2). E. Bioreactor system for growing TE livers. (F) Appearance of the established TE liver (F1) and liver injury model (F2).

Rats were fasted for 24 h and deprived of water for 4 h before the procedure. The rats were anaesthetized with 10% chloral hydrate. After disinfection, the abdominal cavity was opened to expose the liver and portal vein, and an indwelling intravenous needle was inserted through the portal vein. After fixation, a peristaltic pump was turned on at a flow rate of 15–20 mL/min and used to flush the portal vein for approximately 20 min. Then 2% PLA2 enzyme was applied for 10 min followed by precooled HBSS for 20 min, as shown in Figure 1(B). Then, the entire liver was removed, ensuring the integrity of the liver. Subsequently, rat liver scaffolds were ligated and trimmed, and the middle liver lobe was retained. The scaffolds were placed in a special-shaped sterile bottle prefilled with DMEM, connected to a peristaltic pump device, and circulated overnight in an incubator at 37 °C and 5% CO_2_.

### Establishment of CPZ-induced cholestatic TE liver injury

Highly differentiated HepG2 cells were cultured in DMEM supplemented with 10% foetal bovine serum, 100 U/mL penicillin, and 0.1 mg/mL streptomycin. Decellularized scaffolds were recellularized 3 times with 1 × 10^7^ HepG2 cells each time at a rate of 9 mL/min. The perfusion rate was selected based on the hepatic blood flow rate of adult rats during the resting period (85 mL/min/kg). After completion of recellularization, the medium with bile acid mixtures (shown in Table 2) was changed every day for 5 consecutive days. The livers grew after 5 days of culture, and then kuhuang (1 mg/mL) was added on the 7th day of the experimental procedure. Twenty-four h later, on the 8th day, CPZ was added to establish injury, as shown in Figure 1(A). The livers were collected 24 h after CPZ was added, and supernatant samples were collected before and after CPZ administration and examined.

### Analysis of cytotoxicity by the CCK-8 assay

HepG2 cells were suspended at a density of 40–60 cells/µL, and 100 µL of the cell suspension was added to each well of a 96-well plate. The cells were incubated for 24 h, and then kuhuang was added to the culture plate at different concentrations. After 24 h, 10 µL of CCK-8 solution was added to each well according to the manufacturer’s instructions, the culture was incubated for 20 min, the absorbance (optical density [OD]) was measured at 450 nm with a microplate reader, and cell viability was calculated.

### Analysis of ROS, MDA, GSH, and SOD levels

Cells from TE livers were ground, collected in centrifuge tubes and washed 1–2 times with PBS. The precipitated cells were collected by low-speed centrifugation, and then the cells were fully lysed using lysis solution. The supernatants were collected, 500 µL of PBS and 0.5 µL of the fluorescent probe DCFH-DA was added, the supernatants were incubated in a water bath at 37 °C for 40 min and washed twice with PBS, 200 µL PBS was added, the samples were mixed, and a fluorescence microplate reader was used to measure the fluorescence intensity at 480 nm excitation and 525 nm emission. ROS levels in the cells were calculated.

The lysis supernatants were placed in a 96-well plate and reagents and samples were added according to the instructions of the MDA kit. Then, the absorbance of each well was measured at 532 nm using an enzyme marker. Finally, the level of MDA in the cells was calculated.

The supernatants were processed according to the instructions of the GSH kit. The samples were added to a 96-well plate, mixed well, and allowed to stand for 5 min. Then, the absorbance of each well was measured with a microplate reader at 405 nm to calculate the GSH content in the cells.

The samples were added to a 96-well plate according to the instructions of the SOD kit. After mixing and incubating at 37 °C for 20 min, the absorbance was measured with a microplate reader at a wavelength of 450 nm. The concentrations of SOD in the cells were calculated.

### Analysis of AST, ALT, LDH, and ALP levels

Supernatants were collected in a special-shaped bottle after the livers were removed from the machine. The samples were added to a 96-well plate and processed according to the instructions of the corresponding kit. The optical density (OD) of each sample was measured using a microplate reader at the appropriate wavelength, and the enzyme levels in the samples were calculated.

### TBA assay

The TE livers were removed, and C4 collagenase was used to isolate HepG2 cells from the livers. The cells were placed in a 15 mL centrifuge tube, centrifuged at 1000 rpm, and washed 2–3 times with PBS, and the pelleted cells were collected. Then, 150 µL of RIPA lysis buffer was added, and the cells were ultrasonicated to achieve full lysis. According to the instructions of the kit, the samples were placed on 96-well plates. After incubation for 5 min in a 37 °C incubator, the OD was measured with a microplate reader at a wavelength of 405 nm, the plates were incubated in a 37 °C incubator for 2 min, and the OD was measured again with a microplate reader. Finally, according to the appropriate formula and dilution factor, the concentration of TBA in each group of samples was calculated.

### SEM and TEM

The obtained TE livers were fixed for SEM and TEM using 2.5% glutaraldehyde. The samples were then sent to the Electron Microscopy Centre of Peking University Medical School for sample processing. After processing, the samples were observed with a JSM-5600LV scanning electron microscope (JEOL) and a JEM1230PLUS transmission electron microscope (JEOL) at 80 kV. Digital images were acquired using a Gatan Orius SC1000 CCD digital camera with Digital MicroImager 3.11.0 software (Gatan).

### Immunofluorescence analysis of tissue sections

Paraffin sections were dewaxed and hydrated. Antigen repair solution was boiled, and then the sections were placed in the solution, boiled for 10 min, removed, cooled naturally to room temperature, and washed three times with ddH2O. Primary antibodies were diluted with the serum of the secondary antibody host species, and the sections were incubated with primary antibody overnight at 4 °C and washed three times with TBS. The secondary antibody was diluted with the serum of the secondary antibody host species, and the sections were incubated with secondary antibody for 1 h at room temperature and washed three times with TBS. The nuclei were stained with DAPI at room temperature for 10 min. Finally, the slides were sealed, and images were collected using a laser confocal microscope.

### RNA extraction and RT-qPCR

Total RNA was extracted with TRIzol and analysed with a UV spectrophotometer. The RNA concentration was calculated, and then the RNA was treated with DNase. After reverse transcription using a kit from Takara, qPCR was performed on an ABI StepOnePlus machine. The primers used are shown in Table 3.

**Table 3.** Primers used in this study.

Primers		Sequences (5′ – 3′)
GAPDH	F	GGACTCATGACCACAGTCCA
	R	TCAGCTCAGGGATGACCTTG
CYP7A1	F	CAGAACTGAATGACCTGCCA
	R	GGTGCAAAGTGAAATCCTCC
CYP8B1	F	ATGAAGGCTGTGCGAGAG
	R	TCTCTTCCATCACGCTGTC
BSEP	F	TGAGCCTGGTCATCTTGTG
	R	TCCGTAAATATTGGCTTTCTG
MRP2	F	ACAGAGGCTGGTGGCAACC
	R	ACCATTACCTTGTCACTGTCCATGA
FXR	F	ACTGACCTGTGAGGGGTGTA
	R	TGCCCCCGTTTTTACACTTG
DR5	F	ATGGAACAACGGGGACAGAAC
	R	CTGCTGGGGAGCTAGGTCT
PPARγ	F	ACCAAAGTGCAATCAAAGTGGA
	R	ATGAGGGAGTTGGAAGGCTCT

### Western blot analysis

Samples were collected, total protein was extracted, and the protein concentration was determined by the BCA method. A total of 34 g protein per sample was subjected to 30% acrylamide-methylene bisacrylamide gel electrophoresis and transferred via the wet transfer method. After transfer, Lichun red was used to stain the membrane, and protein transfer was assessed. The membranes were incubated with monoclonal antibodies diluted in 5% BSA-TBST overnight at 4 °C. The next day, the membranes were washed, incubated with secondary antibody, shaken for 40 min at room temperature, and washed 3 times with TBST, developed with a luminescent solution, and imaged.

### Identification and analysis of active compounds for docking validation

According to the kuhuang drug certificate of analysis report provided by Lei Yunshang Company and the existing literature report (Wu et al. 2005a), we selected these four monomers (matrine, emodin, sophocarpine and sophoridine) with specific contents as active compounds. The two-dimensional (2D) structures of the compounds were downloaded from the PubChem database as SDF files, optimized with Chem3D software, and saved in × mol2 format. The 3D structures of the target proteins were downloaded from the PDB database (https://www.rcsb.org/), dehydrated and hydrogenated using PyMOL software, and then extracted with AutoDock Vina 1.1.2 software. The 3D structure of the target protein was downloaded from the PDB database, dehydrated and hydrogenated using PyMOL software, hydrogenated with AutoDock Vina 1.1.2 software, charged and combined with nonpolar hydrogen, and saved in × pdbqt format. The active site of the target was determined from the literature, grid box coordinates were set, and the box size was adjusted to 0.1 nm for each small box. Small molecules were docked to the proteins using AutoDock Vina 1.1.2 software.

### Statistical methods

Statistical analysis was performed using GraphPad Prism 5.0 (GraphPad Software Inc., San Diego, CA, USA). The measurement data from the experiments are expressed as the mean ± SEM. SPSS 22 statistical software was used, and one-way ANOVA was used for comparisons between groups and LSD *t*-test was further used for statistical analyses between two independent groups. p* < 0.05, p** < 0.01, and p*** < 0.001 indicate significant differences.

## Results

### Establishment of a DRIC model

**Figure 2.**
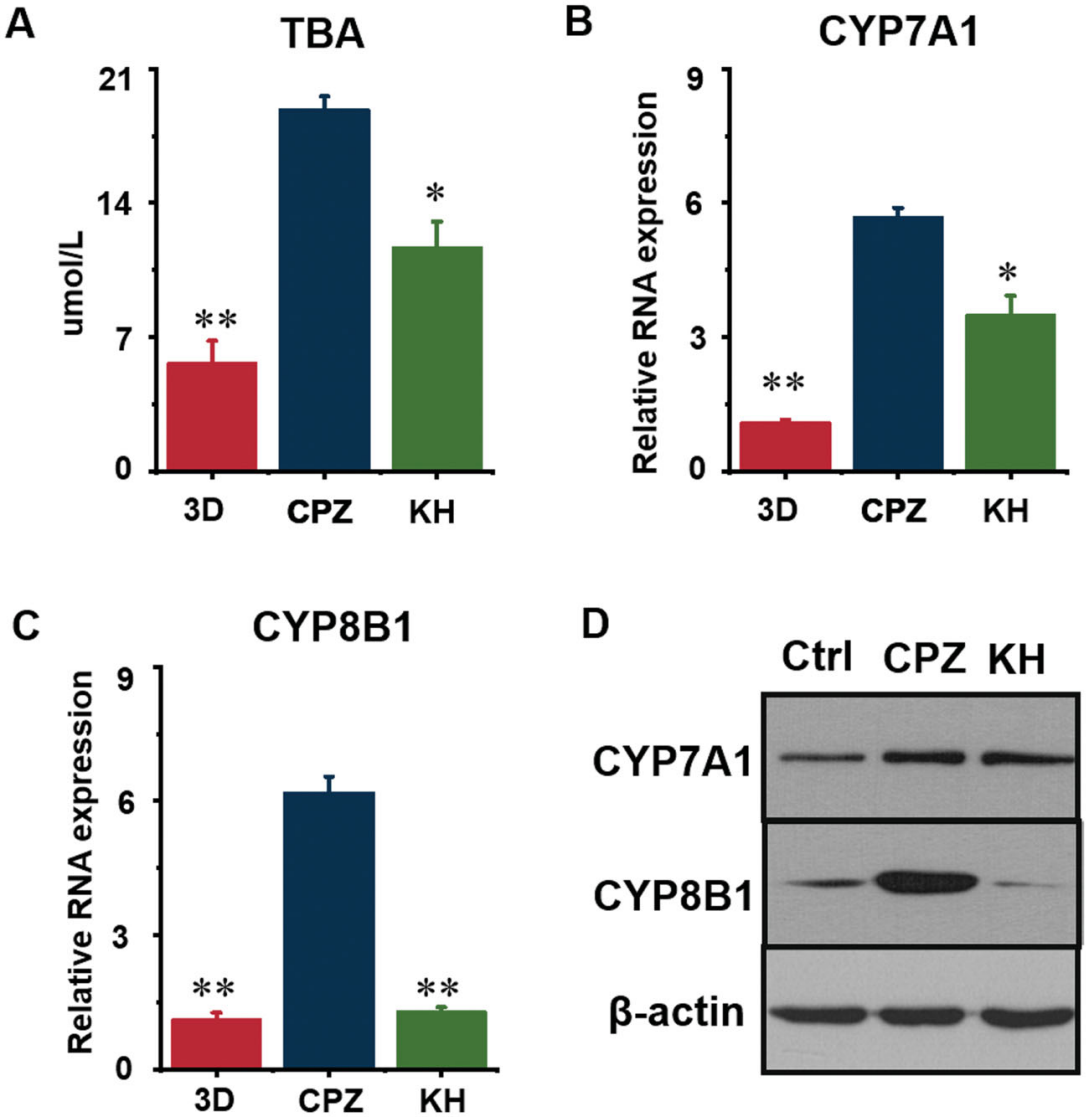
Effect of kuhuang on total bile acids (TBA) and metabolism rate-limiting enzymes in chlorpromazine-induced cholestatic TE liver. (A) The TBA concentrations of 3D, CPZ, and KH group. (B) RNA expression of CYP7A1. C. RNA expression of CYP8B1. D. WB analysis of 3D, CPZ, and KH group. *p < 0.05, **p < 0.01, ***p < 0.001, compared to the 3D CPZ group.

After recellularization, TE livers were continuously perfused with a peristaltic pump in an incubator (Figure 1(E)). Normal control TE livers were cultured in a complete medium. Livers in the 20 μmol/L CPZ-treated group and the 3D group after 7 days in culture are shown in Figure 1(F1 and F2). After CPZ treatment, intracellular TBA levels in the CPZ group were 18.90 ± 0.65 μmol/L, which was a striking increase compared with those of control group (5.67 ± 1.13 μmol/L) (*p* < 0.01, Figure 2(A)).

### Kuhuang injection was essentially non-toxic and protected hepatocytes from CPZ-induced injury in 2D culture

**Figure 3.**
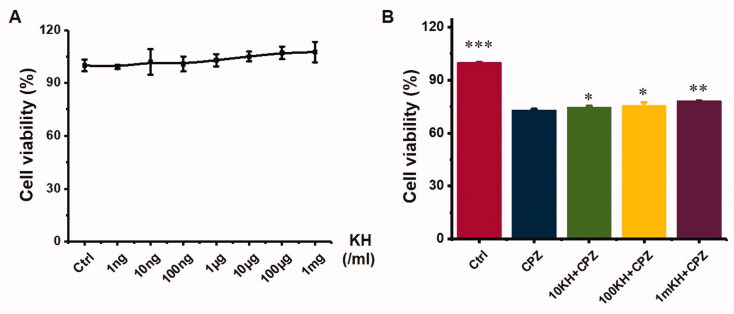
Toxicity test and protective effect of kuhuang injection extract on chlorpromazine-induced HepG2 cell injury. (A) Different concentrations of kuhuang injection extract (1 ng/mL,10 ng/mL, 100 ng/mL, 1 μg/mL, 10 μg/mL, 100 μg/mL, and 1 mg/mL) on cell viability. (B) Protective effect of kuhuang injection extract on chlorpromazine-induced HepG2 cell injury. *p < 0.05, **p < 0.01, ***p < 0.001, compared to the 2D CPZ group.

The cytotoxicity and protective effects of kuhuang were tested in a 96-well plate. Kuhuang had no obvious cytotoxicity at different concentrations (from 1 ng/mL to 1 mg/mL), as shown in Figure 3(A). The protective effect of different concentrations of kuhuang against CPZ-induced injury was further observed, and it was found that kuhuang had a dose-dependent protective effect starting at a dose of 10 µg/mL. Additionally, there was a positive correlation between cell viability with the kuhuang concentration, indicating that kuhuang has a good protective effect on cells, and 1 mg/mL was selected as the experimental drug concentration (Figure 3(B)).

### The protective effect of kuhuang on TE livers injured by CPZ

**Figure 4.**
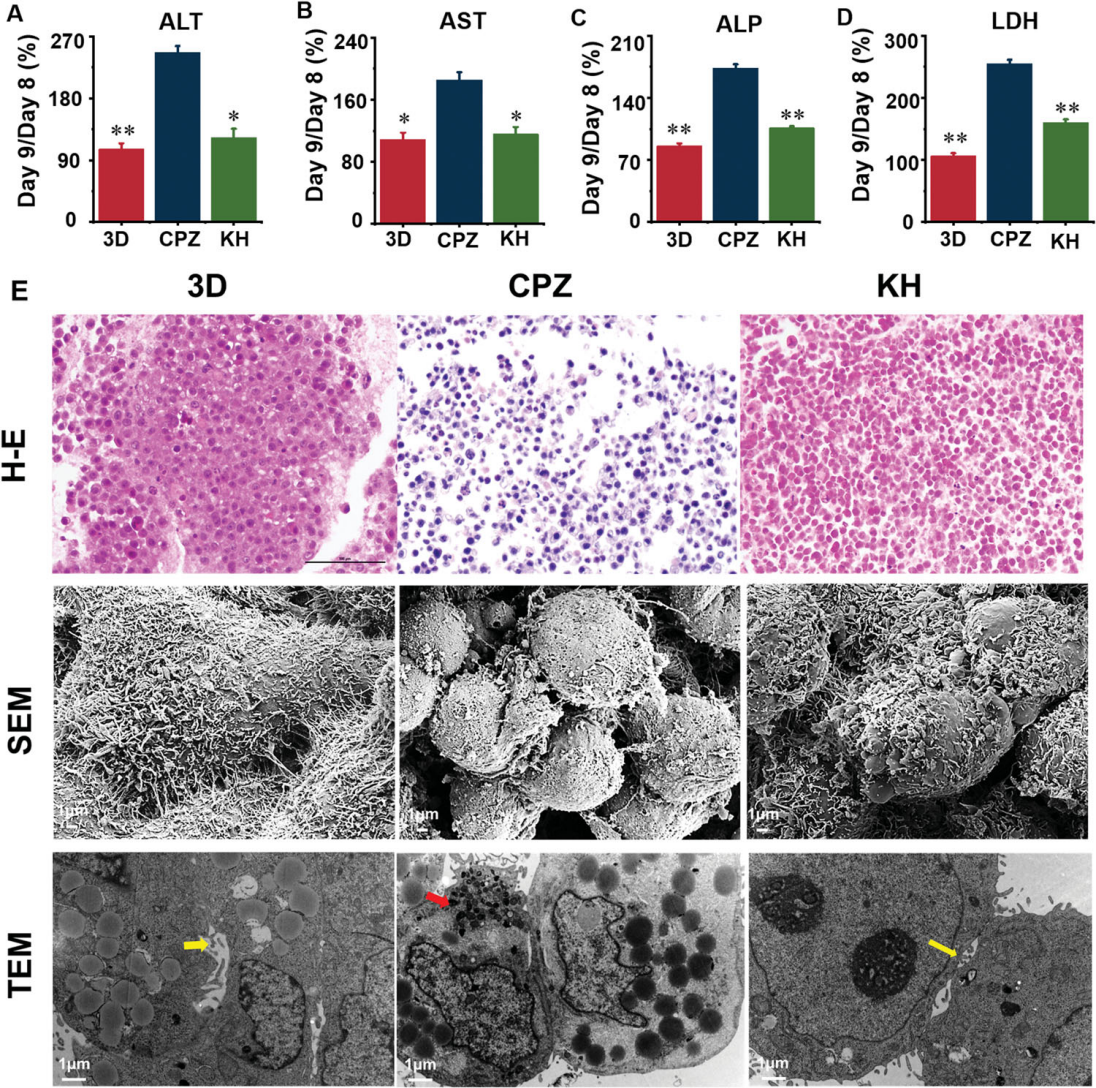
Culture supernatant levels of hepatocyte injury marker and effect of chlorpromazine and kuhuang on histological changes in TE liver tissue. (A) ALT; (B) AST; (C) LDH; (D) ALP; (E) H–E staining, SEM and TEM observation of the 3D, CPZ, and KH (Kuhuang + CPZ) group. Yellow arrow indicates capillary bile duct. Red arrow indicates aggregated bile acid salts.

Administering CPZ to the TE livers caused significant (*p* < 0.05) liver damage and necrosis of cells as evidenced by the elevated serum hepatic enzymes ALT, AST, ALP, and LDH compared with the 3D group (Figure 4(A–D)). Effects of pre-treatment with kuhuang exhibited a significant (*p* < 0.05) decrease in serum activities of ALT, AST, ALP, and LDH (Figure 4(A–D)). HE staining showed that CPZ resulted in more intense staining of the nucleus and reduced staining of the cytoplasm. This indicates that the damage to the cells was significantly alleviated. Additionally, the number of cells was significantly increased in the KH group relative to the CPZ group (Figure 4(E)). SEM revealed that the morphology of the cells was normal, the connection between the cells was tight, the microvilli of the cells were developed, and swelling and rounding of the cells were improved in the KH group compared with the CPZ-treated group (Figure 4(E)). TEM showed that the capillary bile ducts between the cells were absent in the CPZ group but present after treatment with kuhuang. In addition, the injury group showed a significant accumulation of bile salt particles in liver cells (Figure 4(E)). The above data prove that kuhuang exerts a protective effect against drug-induced cholestatic liver injury in this model.

### The effect of kuhuang on the metabolism of bile acids

At the end of culture, the TBA level in the CPZ group was 18.90 ± 0.65 μmol/L, and Kuhuang reduced this level to 11.72 ± 1.31 μmol/L (*p* < 0.05, Figure 2(A)). The classic pathway of bile acid synthesis involves the action of CYP7A1, CYP8B1, and other enzymes. To clarify the molecular mechanisms by which Kuhuang regulates hepatic bile acids accumulation under CPZ treatment, CYP7A1 and CYP8B1 were measured by PCR and WB analysis (Figure 2(B–D)). The results showed that the expression of CYP7A1 and CYP8B1 increased significantly (*p* < 0.01) when CPZ was administered and that Kuhuang reduced the accumulation of bile acids in liver cells by inhibiting the expression of these two rate-limiting enzymes (CYP7A1, *p* < 0.05; CYP8B1, *p* < 0.01) compared to the CPZ group.

### The effect of kuhuang on oxidative stress caused by CPZ

**Figure 5.**
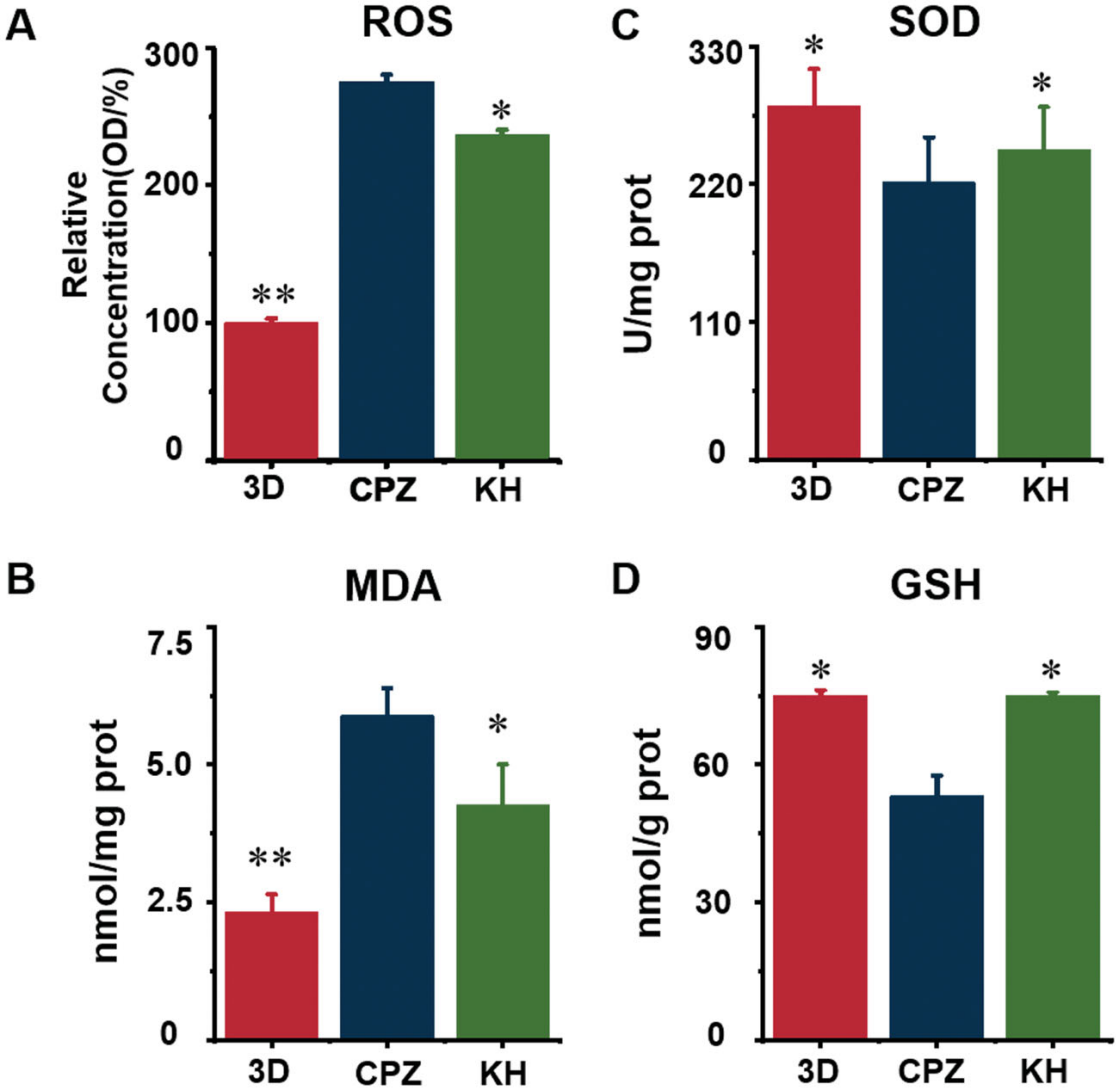
Kuhuang prevented oxidative damage *in vitro*. (A) ROS; (B) MDA; (C) SOD; (D) GSH.

Significantly increased concentrations of ROS and MDA resulted from the addition of CPZ, indicating that CPZ induced oxidative stress in TE livers (*p* < 0.01, Figure 5(A, B)). Moreover, CPZ decreased the SOD and GSH activity level by 22% and 29%, respectively (*p* < 0.05, Figure 5(C, D)). In the KH group, CPZ-induced oxidative stress was significantly reduced (Figure 5). Analysis of ROS and MDA levels showed that kuhuang effectively reduced the production of ROS and MDA in cells upon CPZ-induced damage, thereby protecting cells (*p* < 0.05, Figure 5(A, B)). The GSH and SOD assay results showed that when cells were injured by CPZ, kuhuang effectively increased the content of GSH and SOD in the TE livers (*p* < 0.05, Figure 5(C, D)), thereby increasing the antioxidant capacity of the cells. These results, which are shown in Figure 5, indicate that kuhuang has a protective effect against the oxidative stress response.

### The effect of kuhuang on bile acid export pumps

**Figure 6.**
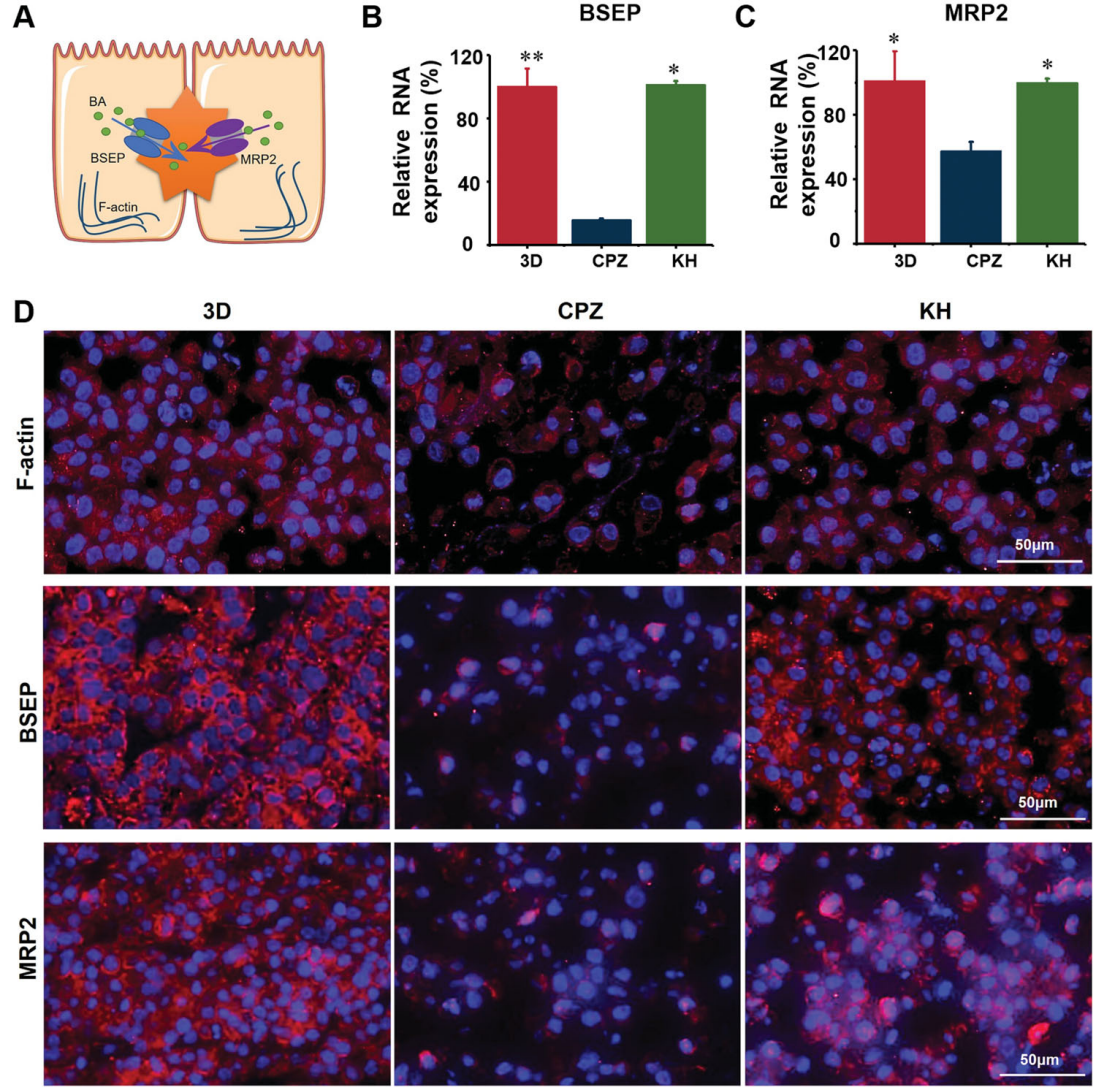
Kuhuang protected MRP2, BSEP, and F-actin damage induced by CPZ. (A) Graphic demonstration of the location of MRP2, BSEP, and F-actin. (B) RNA expression of BSEP. (C) RNA expression of MRP2. (D) Immunofluorescence test of MRP2, BSEP, and F-actin protein expression in different groups.

Under normal physiological conditions, bile acids produced in liver cells are mainly exported by BSEP and MRP2. F-actin is also expressed and helps hepatocytes export bile acids (Figure 6(A)). CPZ induces oxidative stress, which disrupts the integrity of the F-actin cytoskeleton, and structural damage to the F-actin cytoskeleton induces the expression of both BSEP and MRP2. The internalization and endocytosis of bile salt export pump further aggravate the accumulation of bile acids. It was found that the expression of the bile acid export pumps BSEP and MRP2 and F-actin was significantly higher in the kuhuang-treated group than in the CPZ-induced injury group (Figure 6(B–D)). This finding once again confirms that kuhuang exerts protective effects.

### Kuhuang treatment inhibited CPZ-induced oxidative stress via PPAR-γ

**Figure 7.**
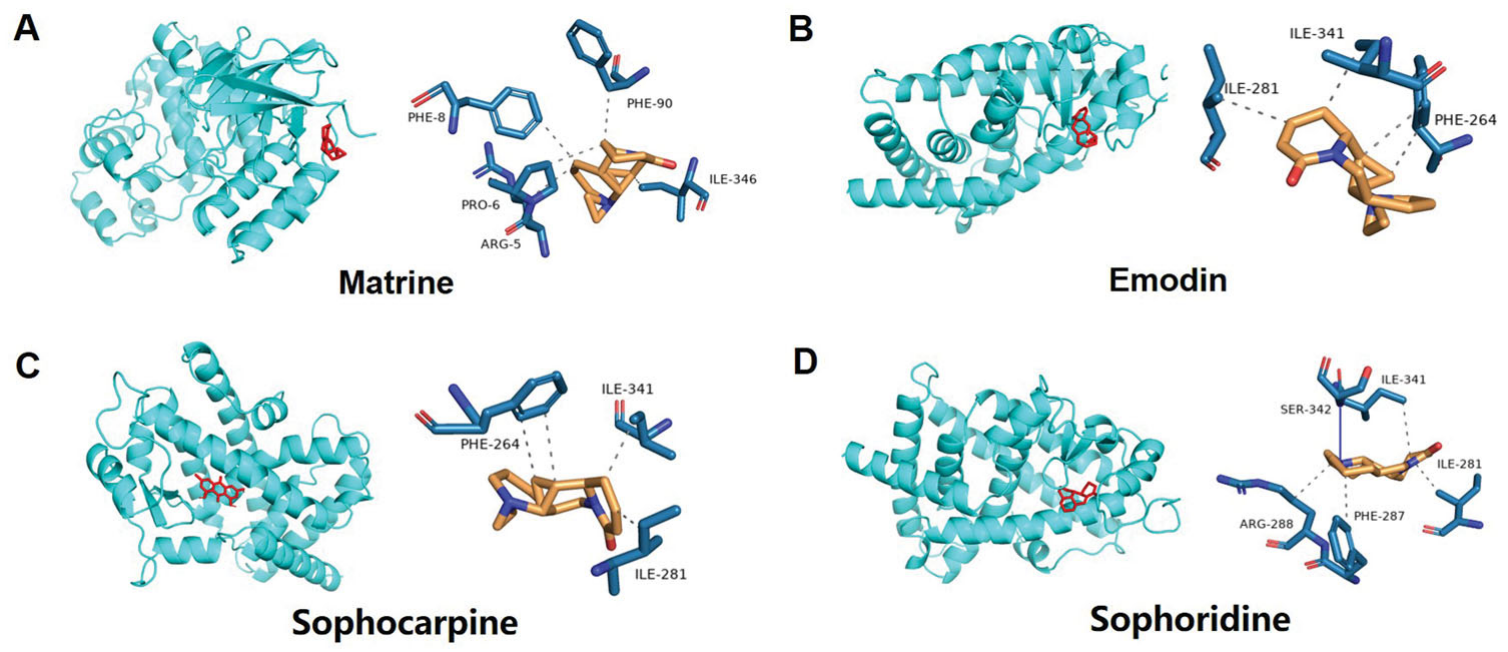
Molecular docking model and visual map of binding site between main components of kuhuang injection and PPARG target. The main components of kuhuang injection are matrine, emodin, sophocarpine, and sophoridine.

According to the kuhuang drug certificate of analysis report and related literature (Wu et al. 2005a), 3 ingredients of Sophorae Flavescentis Radix (matrine, sophocarpine, and sophoridine) and 1 ingredient of Rhei Radix Et Rhizoma (emodin) were identified. Molecular docking between the 4 main components of kuhuang injection and the target PPARG (encodes the protein PPAR-γ) was then performed. The molecular docking binding energies of the 4 components to PPAR-γ are shown in Table 4. It is generally accepted that docking energy values less than −4.25 kcal/mol indicate some binding activity between the two molecules, docking energy values less than −5.0 kcal/mol indicate good binding activity, and docking energy values less than −7.0 kcal/mol indicate strong binding activity. The results showed that all 4 major components were able to bind to the target site of PPARG, and the binding sites are shown in Figure 7.

**Table 4.** The binding energy (BE) of the main components of kuhuang injection and the target molecule of PPARγ (Kcal/mol).

Sources	Compound	MV	BE
Sophora flavescens	Matrine	248.41	–7.7
Radix Rhei Et Rhizome	Emodin	270.24	–8.5
Sophorae Flavescentis Radix	Sophocarpine	246.35	–7.8
Sophorae Flavescentis Radix	Sophoridine	248.36	–7.8

**Figure 8.**
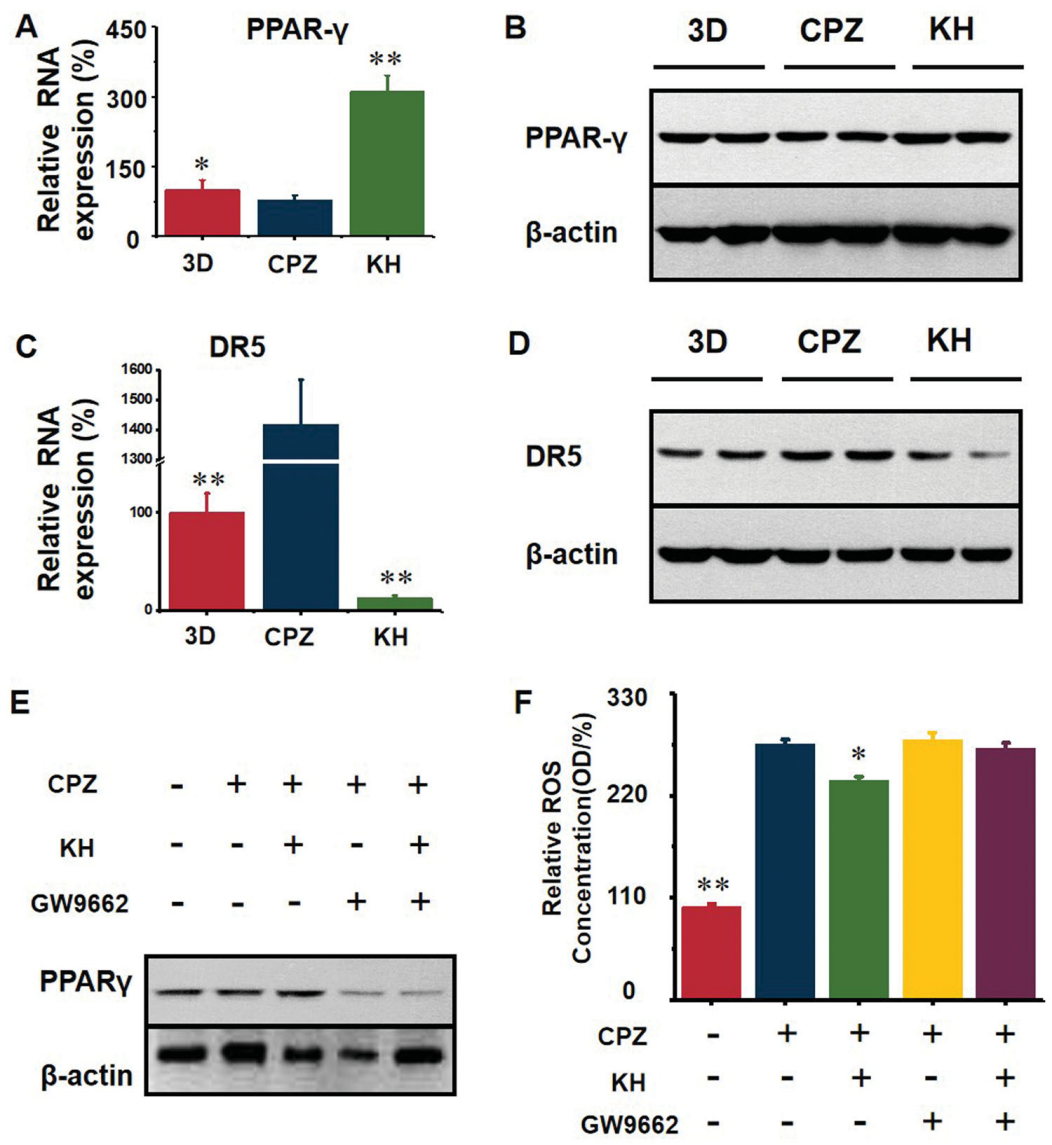
Kuhuang could have an antioxidant effect and protect cell death via activating PPARγ. (A) RNA expression of PPARγ in different groups (3D, CPZ, KH). (B) WB analysis of PPARγ in different groups (3D, CPZ, KH). (C) RNA expression of DR5 in different groups (3D, CPZ, KH). (D) WB analysis of DR5 in different groups (3D, CPZ, KH). (E) WB analysis of PPARγ in different groups (3D, CPZ, KH, CPZ + GW9662, and KH + GW9662). GW9662 is the inhibitor of PPARγ. (F) ROS concentration in different groups (3D, CPZ, KH, CPZ + GW9662, and KH + GW9662).

After CPZ-induced injury, oxidative stress resulted in the downregulation of PPAR-γ expression (*p* < 0.05, Figure 8(A, B)) compared to the 3D group, and after kuhuang administration, the expression of PPAR-γ increased significantly (*p* < 0.01, Figure 8(A, B)) compared to the CPZ group, indicating that kuhuang exerted a protective effect by inhibiting oxidative stress via an increase in PPAR-γ expression, ultimately leading to a decrease in the gene and protein expression of the death receptor DR5. The above results indicate that kuhuang exerts an inhibitory effect by increasing PPAR-γ expression and reducing DR5 expression (*p* < 0.01, Figure 8(D)) compared to the CPZ group. Then, GW9662 (a PPAR-γ inhibitor) was used to confirm that kuhuang-induced inhibition of oxidative stress is dependent on PPAR-γ. Our results showed that kuhuang injection extract treatment did not further influence ROS overproduction following treatment with GW9662 in the presence of CPZ (Figure 8(F)).

## Discussion

DRIC is an important clinical problem that can lead to drug withdrawal and discontinuation, resulting in significant economic losses (Sundaram and Björnsson 2017). Due to differences in bile acid metabolism among species, animal models cannot simulate DRIC well (Petrov et al. 2018; McGill and Jaeschke 2019). However, the strategies for preclinically predicting a drug’s capacity to induce DRIC are currently limited to measuring the compound's potential to inhibit BSEP (Cheng et al. 2016; Gijbels et al. 2019). However, the manifestation of DRIC is often complex and multifactorial. Importantly, 3D interactions between cells and structures with noncellular components occur in the human body. However, classic 2D monolayer cell culture models cannot mimic these interactions between cells and components or matrices (Kuna et al. 2018). In the past decade, 3D cell culture systems, such as organoids and spheroids, have been developed to mimic the hepatic structure and cellular interactions *in vitro* (Sato et al. 2021). These 3D cell culture models cannot fully simulate the liver microstructure and circulatory perfusion system, which are very important for maintaining hepatocyte polarity and function. Therefore, new *in vitro* models that can be used to comprehensively assess the cholestatic risk of compounds in a physiologically relevant liver model are needed. We constructed a 3D TE liver model in the early stage, which can be used to better simulate drug metabolism by the liver and pathology (Wu et al. 2018; Han et al. 2019; Zhang et al. 2021). Because the model utilizes human hepatocytes, species variability is not an issue, and the model can be used to simulate DILI.

CPZ is a well-known cause of acute cholestatic liver injury (Todorović Vukotić et al. 2021). Numerous instances of apparent CPZ-induced acute liver injury, which is estimated to occur in 1 in 500 individuals exposed to CPZ, have been reported in the literature. Therefore, we used CPZ to injure TE livers and establish DRIC in this study. Increasing evidence shows that oxidative stress plays an important role in liver injury in rodents and humans with cholestatic liver disease (Anthérieu et al. 2013; Hendriks et al. 2016; Burban et al. 2018). In CPZ-induced cholestasis, oxidative stress can impede the secretion of bile acids by disrupting the cytoskeletal protein F-actin in hepatocytes and inducing internalization of BSEP and MRP2, causing intracellular accumulation of toxic bile acids, which induces DR5 expression and thus leads to activation of the death receptor signalling pathway. PPAR-γ is a ligand-inducible transcription factor of the nuclear hormone receptor superfamily (Ahmadian et al. 2013). It is expressed in hepatocytes and exerts antioxidant effects by inducing the expression of antioxidant enzymes through binding to elements in the promoters of antioxidant genes (Sheng et al. 2020; Xiang et al. 2021). Furthermore, docking software predicted that 14 main compounds of kuhuang injection had binding domains for PPAR-γ. In this study, we found that kuhuang prevented oxidative stress at least partially via the PPAR-γ signalling pathway. The PPAR-γ antagonist GW9662 was administered in combination with kuhuang injection extract, and the results showed that inhibition of PPAR-γ markedly alleviated the ability of Kuhuang injection to alleviate oxidative stress induced by CPZ, indicating that PPAR-γ is required for the effect of kuhuang and that PPAR-γ activation is a potential therapeutic strategy for cholestasis. A previous study revealed that thiazolidinediones, agonists of PPAR-γ, increase the expression of BSEP. Xiang et al found that tectorigenin, a partial agonist of PPAR-γ, alleviates cholestasis in an experimental mouse model (Xiang et al. 2021). These data indicate that activating PPAR-γ may be an effective target for the treatment of cholestatic liver disease. Additionally, we propose that Kuhuang can activate PPAR-γ, thus alleviating oxidative stress induced by CPZ, which can lead to disruption of F-actin and thus cause endocytosis of BSEP and MRP2, reducing the accumulation of toxic bile acids in cells, downregulating the expression of the apoptotic protein DR5 and protecting hepatocytes. This may also be the molecular mechanism by which kuhuang exerts its antioxidant and anti-injury effects in the cholestatic drug liver injury model.

## Conclusions

**Figure 9.**
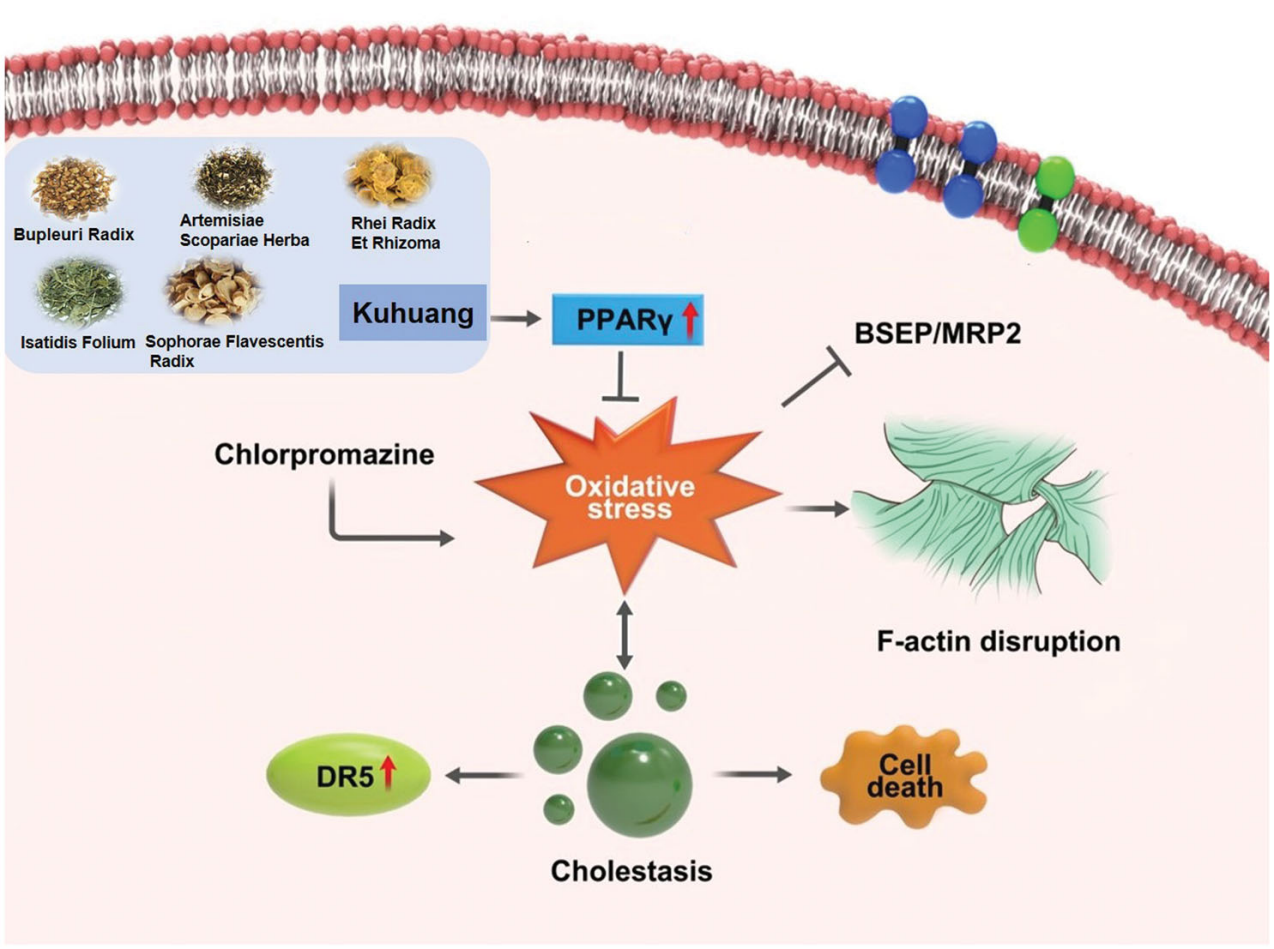
Kuhuang alleviates CPZ-induced TE liver injury by activating PPARγ dependent antioxidant pathway.

This study shows that 1 mg/mL kuhuang injection extract exerts a significant anticholestatic effect, possibly by activating PPAR-γ signalling and reducing the internalization of MRP2 and BSEP caused by oxidative stress-induced disruption of F-actin, thereby alleviating CPZ-induced intrahepatic cholestasis and reducing the expression of DR5 and cell death (Figure 9). These findings are expected to provide the basis for the clinical use of kuhuang injection in the treatment of drug-induced cholestatic liver injury.
